# Effect of different surface treatments on color and translucency of two different ceramic materials (an invitro study)

**DOI:** 10.1186/s12903-026-08920-4

**Published:** 2026-06-29

**Authors:** Mohamed Gamal Hamdy Elbadry

**Affiliations:** Fixed Prosthodontics Department, Faculty of Dentistry, Minia University, Misr Aswan Agricultural Rd., Ard Shalaby, Minia, Egypt

**Keywords:** Ceramics, Laser therapy, Hydrofluoric acid, Sandblasting, Translucency, Spectrophotometry

## Abstract

**Background:**

Surface treatment enhances bonding to ceramic restorations but may alter their optical properties. Lithium disilicate and zirconia respond differently to surface treatments due to variations in their microstructure. This study evaluated how sandblasting, hydrofluoric acid with silane, and laser treatment affect the color stability and translucency of these ceramics.

**Aim of the study:**

This study aimed to evaluate the individual and combined effects of ceramic material type and surface treatment on the color stability and translucency of lithium disilicate and zirconia ceramics.

**Materials and methods:**

Forty samples were constructed in the form of discs form lithium disilicate and zirconia ceramic (*n* = 20 for each). Samples from each material were divided into four subgroups according to the surface treatment used as following: (Ctrl); control with no surface treatment, (S.B); sandblasting using AL_2_O_3_, (H.F); hydrofluoric acid etching with silane coupling agent, (L.A); laser treatment. Each subgroup from each material was undergoing color and translucency test using spectrophotometer. The data were statistically analysed using a Two-Way ANOVA, followed by multiple pairwise comparisons using Tukey’s HSD for each individual factor.

**Results:**

Among the tested surface treatments only laser showed color change within the clinical accepted range with lithium disilicate ceramic (1.04 ± 0.15), also hydrofluoric acid etching with silane application showed that with zirconia (0.41 ± 0.10). Sandblasting showed the lower translucency value with lithium disilicate and zirconia ceramic with a significant difference with the control samples (*p* value ˂0.001).

**Conclusions:**

Surface treatments affected the optical properties of both ceramics in a material-dependent manner. Laser conditioning preserved color acceptably in lithium disilicate, while hydrofluoric acid with silane did so for zirconia. Sandblasting significantly reduced translucency in both materials, indicating its potential to compromise esthetic outcomes.

## Background

+The increasing demand for esthetic rehabilitation has positioned ceramic restorations as a widely accepted treatment option for diverse clinical indications, including enamel hypoplasia, fluorosis, tetracycline staining, and diastema closure. IPS e.max CAD, a machinable lithium disilicate glass ceramic developed for CAD/CAM applications, has become a material of choice owing to its superior optical properties and ability to closely mimic natural tooth structure. To optimize the adhesion between ceramic restorations and resin cements, several surface conditioning protocols have been advocated, most notably hydrofluoric acid etching combined with silane application, as well as air-particle abrasion using aluminum oxide particles. More recently, various laser systems have been introduced as alternative surface treatment modalities to further enhance resin–ceramic bond strength [1–4]. 

Translucent zirconia is regarded as an esthetic monolithic material with a high fracture resistance [5]. These qualities enable the material to be employed in minimally invasive repairs [6, 7]. However, highly translucent zirconia is considered a polycrystalline structure that is chemically inert which makes its adhesion to the tooth structure troublesome compared with silica-based ceramics. A strong bond between translucent zirconia and resin cement is absolutely required; therefore, the inner surface of the restoration should be surface treated for mechanical and chemical bonding [8]. A number of surface treatments, including laser irradiation and airborne particle abrasion, have been suggested [9]. 

Optical features like color stability and translucency should be taken into account during the selection of ceramic restorative materials to maintain esthetics [10]. 

The success of dental restoration is measured when its optical properties mimic those of natural dentition [11]. Color of dental ceramics is crucial for the success and longevity of the restorations. It is greatly influenced by surface roughness [12]. Surfaces having varied degrees of roughness cause changes in hue, chroma and value. Modifications of surface affect the value mostly, the smoother the surface, the higher its value [13, 14]. Another crucial factor for the success and longevity of the esthetic restorations is translucency. Simply the translucency relies on the behaviour of the incident light when it falls on an object in the form of transmission, reflection, refraction, scattering and absorption. The object is considered translucent if the light is scattered and most of it is diffusely transmitted, while the object is opaque if most of light is absorbed and diffusely reflected [15]. These optical qualities could be altered by the material surface treatments necessary for bonding [16]. 

An objective measurement of color and translucency of dental materials can be done using special tool called spectrophotometer [17]. Spectrophotometers evaluate the color according to the CIE Lab color scale relative to the International Commission on Illumination. This system defines color in terms of three coordinate values (L*, a*, and b*) which locate the color of material within a 3D color space. The L* represents the brightness of an object, the a* represents the red/green chroma and the b* represents the yellow/blue chroma [18]. 

So, the aim of this study was to evaluate the effect of different surface treatments on color and translucency of lithium disilicate glass-based ceramic versus zirconia ceramic. The primary objective of this in vitro study was to evaluate the individual and combined effects of ceramic material type and surface treatment on the color stability and translucency of lithium disilicate and zirconia ceramics.

The null hypothesis of this study suggested that there would be no significant influence of surface treatment on color and translucency of lithium disilicate glass-based ceramic and zirconia ceramic.

## Materials and methods

This study received ethical approval from the Research Ethics Committee of the Faculty of Dentistry, Minia University, Egypt, (Commitee No. 121, Decision No. 1184, Date: 25/08/2025).

A power analysis was conducted using G*Power software (version 3.1.9.7; Heinrich Heine University, Düsseldorf) to determine the optimal sample size, with an alpha level of 0.05, a statistical power of 80%, and an effect size of 1.41 derived from previous studies [19, 20]. The minimum required sample size was calculated to be 16 specimens. To avoid overestimation of the effect size derived from prior literatures, the sample size was increased to 40 specimens. The effect size used for the initial sample size estimation was based on preliminary observations and may overestimate the true clinical effect. Therefore, the present investigation should be interpreted as an exploratory in vitro study. Nevertheless, post hoc achieved power analysis based on the observed factorial ANOVA outcomes demonstrated adequate statistical power for the investigated comparisons.

### Specimens’ preparation

#### Lithium disilicate discs construction

High translucent block of CAD/CAM esthetic ceramic material which was lithium disilicate (IPS e.max CAD, Ivoclar Vivadent AG, USA) with A2 shade, was used. First, the block was prepared by circulation and roundation to reshape the blocks into diameter of 10 mm by a metal turning machine. The block with the recommended diameter was installed on a specially designed metal using self-acrylic resin (IMICRYL, Konya, Turkey). The block was cut to produce discs with the recommended thickness which was 1 mm by isomet 4000 micro saw (Buehler, USA). The thickness of the samples was assessed using a digital micrometer (Praecimeter S, 0.01 mm; Renfert Gmb H, Hilzingen).

**Fig. 1:**
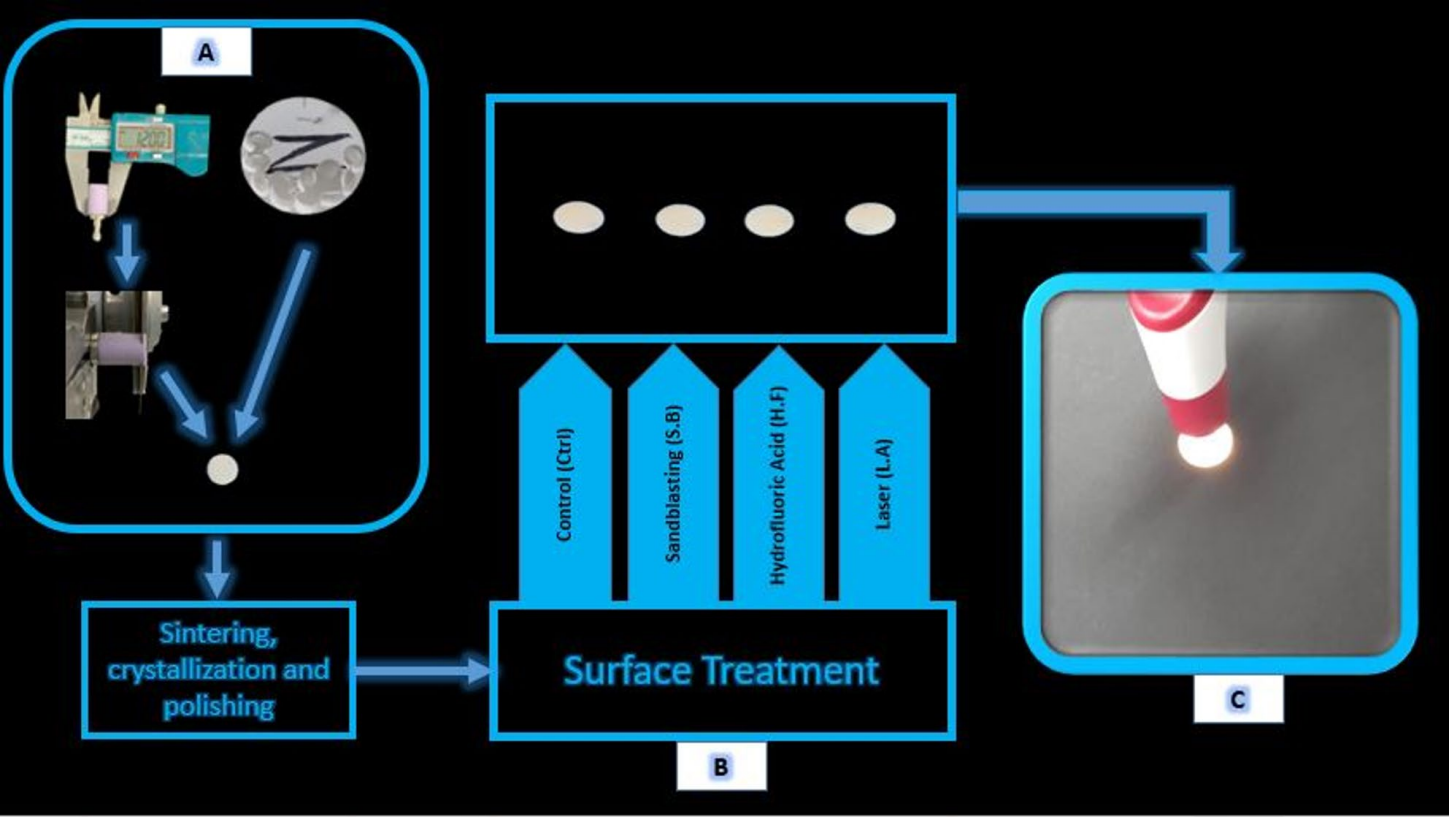
Schematic representation of the experimental workflow used in the study. **A**: Zirconia and lithium di-silicate discs’ construction; **B**: surface treatment subgroups; Ctrl: not subjected to any surface treatment; H.F: subjected to hydrofluoric acid etching and silane coupling agent; S.B: subjected to sandblasting using AL2 O3; L.A: subjected to laser; **C**: Spectrophotometric analysis

After cutting the lithium disilicate discs were placed in a furnace for crystallization using TABEO furnace (MIHM-VOGT GmbH, Stutensee Blankenloch, Germany) The samples were first pre-dried at 403 °C for 6 min and the heating temperature was then increased at a rate of 90 °C/min until reached 820 °C and held for 10 min, then the temperature was then increased at a rate of 30 °C/min until reached 840 °C and held for 7 min. At this specific temperature, the metasilicate dissolves while the lithium disilicate undergoes crystallization. After crystallization, the lithium disilicate discs were finished using aluminum oxide sandpaper (SUNBurst, Korea) and cleaned with an ultrasonic cleaner (Cavitron, Dentsply Intl, York, Pa) (Fig. 1.A).

#### Zirconia discs construction

The discs were designed using Cinema 4D Broadcast R13 (MAXON, Germany) and were planned to have a diameter of 10 mm and a thickness of 1 mm from 5 yttria UTML zirconia ceramic (Zolid FX Multilayer, Amann Girrbach) with A2 shade.

A CAD file was prepared for disc samples with final dimension of 10 diameter x1 mm thickness and converted into STL file. With the specimen STL file, presintered discs were fabricated by using CAD/CAM machining from the respective zirconia blanks. imes icore five-axis dental CAD/CAM milling system (CORTEC 250i, imes icore, GmbH, Eiterfeld, Germany) was used to fabricate the discs. The discs milling procedure was conducted where the size of each disc was larger than the required dimension by 25% to compensate for sintering shrinkage.

Sintering of zirconia discs was done according to manufacturer`s instructions. The firing cycle was done at TABEO sintering machine (MIHM-VOGT GmbH, Stutensee Blankenloch, Germany). The firing cycle was controlled as followings: the temperature was raised to 950°c for 1.5 h and maintained for 3 h, and then raised up to 1550°c for 1.5 h and maintained for 3 h.

The thickness of the samples was assessed using a digital micrometer) Praecimeter S, 0.01 mm; Renfert Gmb H, Hilzingen, Germany) after sintering.

The discs were polished using zirconia polishers (Zirkonzahn, Gais, Italy) A standardized polish was performed where the discs were sequentially machine polished with wet 320, 400, 600, 800, 1000 and 1200 of silicone carbide papers (Microdent, 3R Ind, Com.Brazil). The sequence of polishers used was following the manufacturer`s instructions.

Polishing was done with stroke movements for 30 s for each tool by a single operator, following the sequence and speed recommended by the manufacturer [21]. 

After polishing, the thickness of the samples was assessed using a digital micrometer) Praecimeter S, 0.01 mm; Renfert Gmb H, Hilzingen, Germany) (Fig. 1.A).

### Grouping of the specimens

All 20 specimens from each ceramic material were randomly allocated into surface treatment subgroups. Randomization was performed using an online statistical tool (Randomizer.org). Each specimen was given a unique identifier, and block randomization was used to ensure balanced distribution across all subgroups within each material type.

**Fig. 2:**
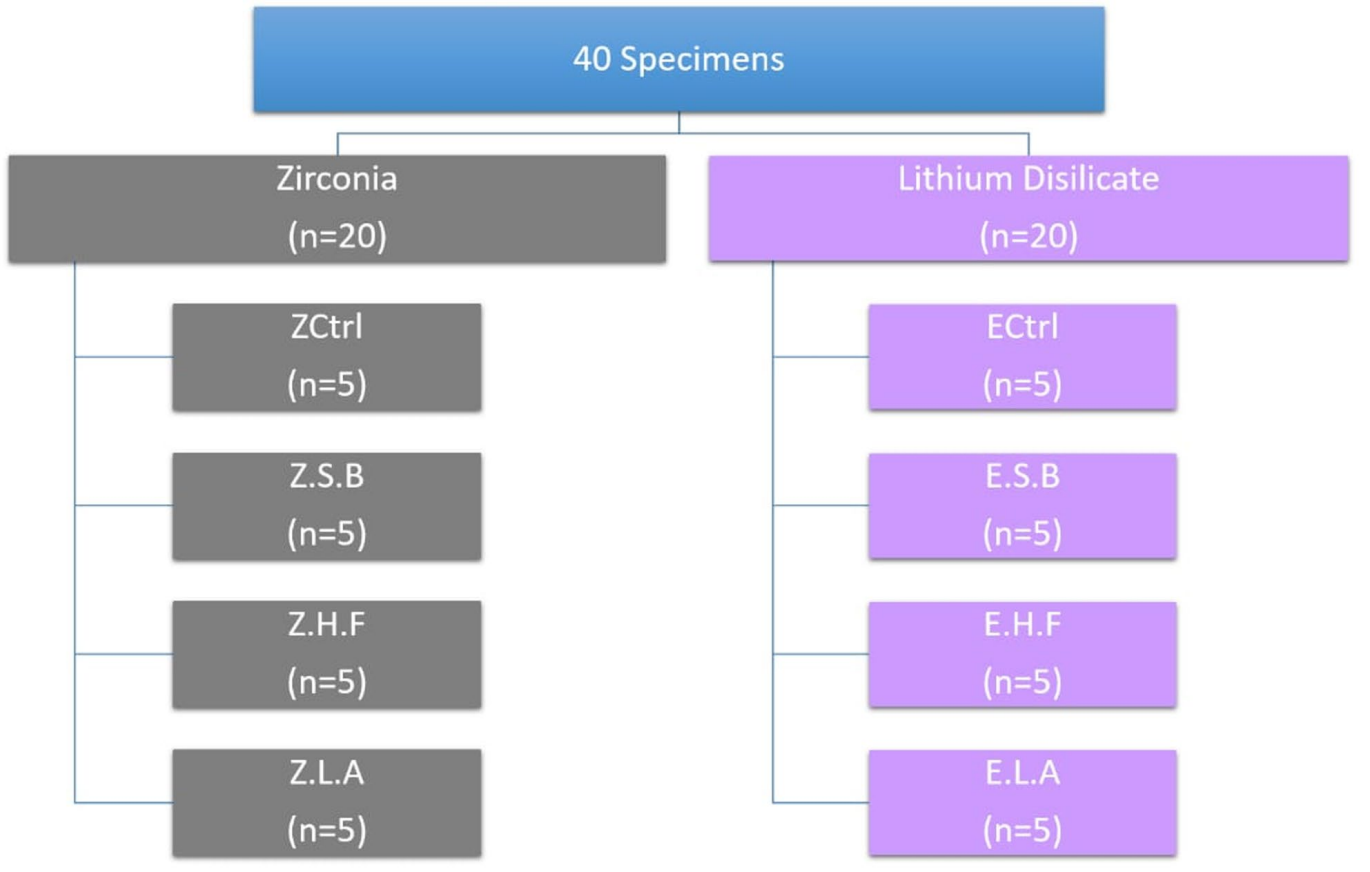
Flowchart of the study design

The allocation sequence was concealed, and the assignments were only revealed at the time of surface treatment to prevent allocation bias. The study included the following four subgroups:

- Ctrl: not subjected to any surface treatment C.
- S.B: subjected to sandblasting using AL_2_ O_3_ SB.
- H.F: subjected to hydrofluoric acid etching and silane coupling agent HF.
- L.A: subjected to laser treatment (Fig. 2) LA.

### Surface treatment

The discs from each material were divided into four subgroups according to the type of surface treatment used as following:

- The first subgroup: was used as a control and was not subjected to any surface treatment.
- The second subgroup: was subjected to sandblasting.
- The third subgroup: was subjected to hydrofluoric acid etching and silane coupling agent.
- The fourth subgroup: was subjected to laser treatment (Fig. 1.B).

### Sandblasting of lithium disilicate discs and zirconia discs

Sandblasting of the lithium disilicate and the zirconia discs was done using 110 μm aluminum oxide particles (Lvdi Centre, Daxue South Road, ZhengZhou City, Henan Province, China) using Renfert air blasting machine (Renfert GmbH-Hilizingen, Germany) at a maximum pressure of 2.0 bar for a short duration (10 s) from a distance of approximately 10 mm. Only sandblasting was done for the surfaces to be bonded and then cleaning the specimen afterward using ultrasonic cleaning (Cavitron, Dentsply Intl, York, Pa) followed by drying with clean oil-free air [22, 23]. 

### Hydrofluoric acid etching of lithium disilicate glass-based ceramic discs and zirconia discs

For, lithium disilicate glass-based ceramic, first cleaning the intaglio surface was performed then hydrofluoric acid etching gel (Ultradent, USA) was applied for 20 s. Thoroughly rinsing the gel with water and dry with oil-free compressed air was done. Next, a silane primer (Ultradent, USA) was applied to the etched surface and allowed it to air dry.

For zirconia ceramic, the samples were ultrasonically cleaned in distilled water for 10 min, then were immersed in 9.5%HF (Bisco Porcelain Etch, USA) for 3 h. After immersion the samples were ultrasonically cleaned in water for 10 min [24]. Then, a layer of all bond universal (Bisco, Schaumburg, Illinois, USA) was applied followed by application of a light jet of air. The adhesive was light‑cured for 20s with a light curing unit at an intensity of 1000 mW/cm2. The light intensity was monitored by a radiometer [25, 26]. 

### Laser treatment of lithium disilicate glass based ceramic discs and zirconia discs

Zirconia discs were irradiated with Er: YAG laser (Fotona; AT Fidelis, Ljubljana, Slovenia). Parameters settings of used device were: wavelength: 2940 nm; frequency: 10 Hz; energy: 200 mJ; output power: 2 W for duration of 10s. A dental hand piece with a sapphire cylindrical fiber-optic tip was used perpendicular to the disc surface with water irrigation. Discs surfaces were processed with the application tip in slight contact, with an up and down motion [27, 28]. Before and after surface treatment, the ceramic discs were ultrasonically cleaned (Cavitron, Dentsply Intl, York, Pa) in a distilled water for 10 min then dried with an oil-free air spray [29, 30]. 

After each treatment, the thickness of the samples was assessed using a digital micrometer) Praecimeter S, 0.01 mm; Renfert Gmb H, Hilzingen, Germany).

### Color measurement

Color measurements were carried out by one operator who was not aware of the groups to remove operator bias. Spectrophotometer (Cary 5000, USA) was used to record the initial color measurements before surface treatments. The average of the three readings were taken against a white background (Fig. 1.C).

All samples were measured with a spectrophotometer (Cary 5000, USA) to determine the color differences (ΔE00) after surface treatments. The CIELAB coordinates (L*, a*, and b*) were recorded at baseline and after exposing all the samples to different surface treatments [31, 32].

The specimen was surrounded by a diffused grey backdrop in accordance with the ISO 7491:2000 criteria for color stability tests, an international standard that specified a method for determining the color stability of dental materials [31]. 

The ΔE_00_ of all specimens were analyzed with the color measurement before and after the surface treatments according to the CIEDE2000 color-difference equation [31]:

$${{\Delta}\mathrm{E}}_{00}=\sqrt{{\left(\frac{{\Delta}{\mathrm{L}}^{{\prime}}}{{K}_{L}{S}_{L}}\right)}^{2}+{\left(\frac{{\Delta}{C}^{{\prime}}}{{K}_{C}{S}_{C}}\right)}^{2}+{\left(\frac{{\Delta}{H}^{{\prime}}}{{K}_{H}{S}_{H}}\right)}^{2}+{R}_{T}\left(\frac{{\Delta}{C}^{{\prime}}}{{K}_{C}{S}_{C}}\right)\left(\frac{{\Delta}{H}^{{\prime}}}{{K}_{H}{S}_{H}}\right)}$$

where ΔL, ΔC and ΔH are the differences in lightness, chroma and hue for a pair of samples inCIEDE2000 and RT is a function (the so-called rotation term) that accounts for the interaction between chroma and hue differences in the blue region.

SL: lightness, SC: chroma and SH: hue.

KL: lightness, KC: chroma and KH: hue [33].

The clinical acceptability threshold (AT) for (ΔE_00_) defines the point at which observers accept a color difference between two items, typically in dentistry. It is generally accepted that a ΔE_00**≤**_1.8 is excellent, while values between 1.8 and 2.8 are often considered clinically acceptable in dental studies. Values above 5 are usually deemed unacceptable in dermatological matching.

### Translucency test before surface treatment

Following the completion of the sintering process, the lithium disilicate glass based ceramic discs and the zirconia discs were let to cool at ambient temperature to obtain baseline data. In this stage, the baseline color of each sample was measured by a spectrophotometer device (Cary 5000, USA).

The CIE L*, a*, and b* coordinates were acquired by employing a light source illumination (D65) that replicates typical daylight conditions.

The spectrophotometer apparatus was subjected to calibration processes against white and black backgrounds. The measurements were replicated three times and the average of the measurements was taken into account.

The determination of TP values was achieved by employing the subsequent formula [34]:

  

$$Tp={[{\left(\mathrm{Lb}\:-\mathrm{Lw}\right)}^{2}+{\left(\mathrm{ab}\:-\mathrm{aw}\right)}^{2}+{\left(\mathrm{bb}\:-\mathrm{bw}\right)}^{2}]}^{\raisebox{1ex}{$1$}\!\left/\:\!\raisebox{-1ex}{$2$}\right.}$$

The variable L* denotes the attribute of lightness or the black/white dimension of a color.

a* signifies the red‑green axis, and b* represents the yellow‑blue axis.

The color coordinates B and W are used to express color values in relation to the black and white background (Fig. 1.C).

### Translucency test after surface treatment

After subjecting the lithium dislicate ceramic discs and the zirconia discs to different surface treatments (control, sandblasting, hydrofluoric acid etching with silane application, laser treatment) from one side only (the unglazed surface), translucency test was done the same as before surface treatment.

## Statistical analysis

Descriptive statistics were reported as means and standard deviations for all material–treatment subgroups. The Shapiro–Wilk test was used to assess normality, and Levene’s test verified homogeneity of variances. Both assumptions were satisfied. The effects of the two independent factors ceramic material type (lithium disilicate, zirconia) and surface treatment (control, sandblasting, hydrofluoric acid etching with silane, and laser) on color change (ΔE₀₀) and translucency were analyzed using a two-way analysis of variance (ANOVA). When significant main or interaction effects were detected, Tukey’s HSD test was applied for multiple pairwise comparisons among subgroup levels. A significance level of *p* < 0.05 was adopted for all statistical tests.

## Results

**Fig. 3:**
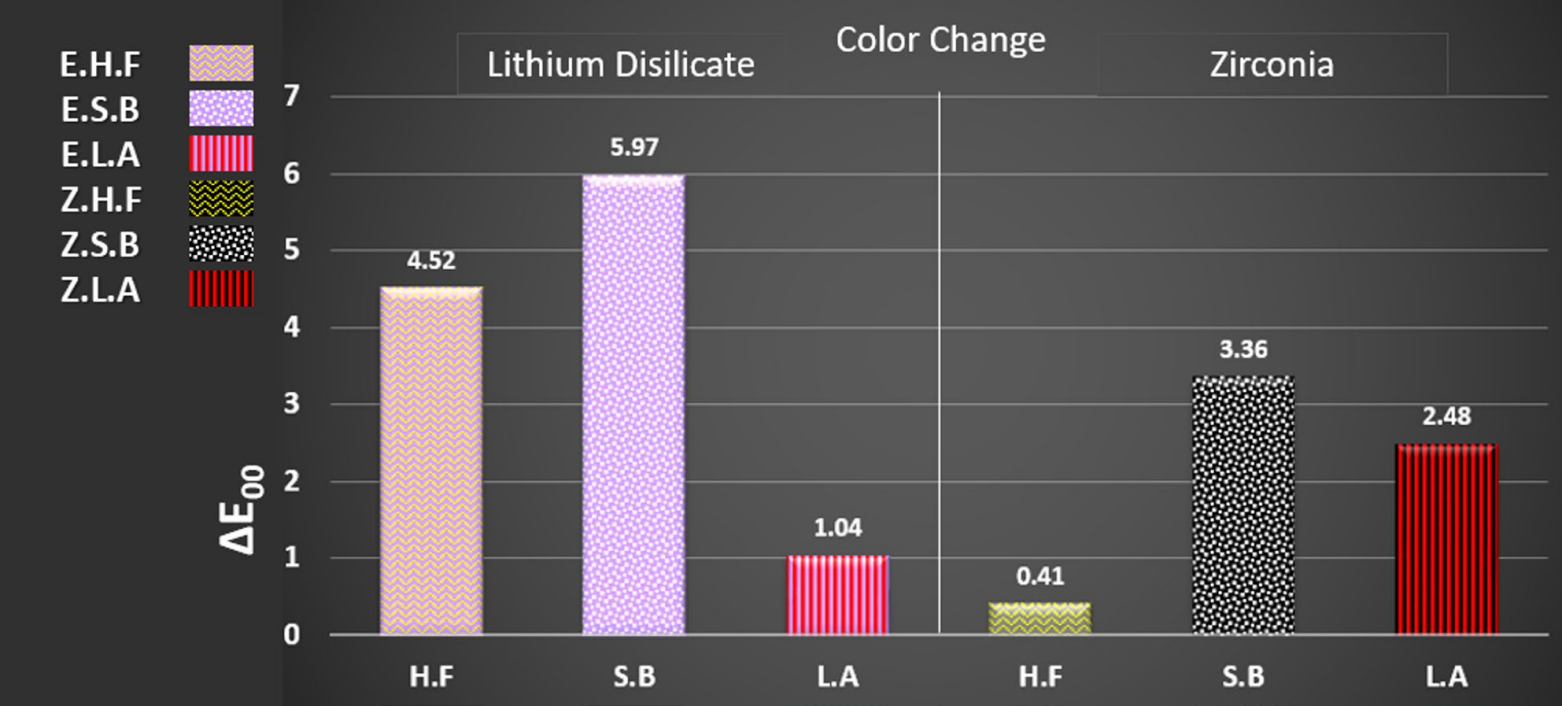
Bar graph presenting the color change of different subgroups. ΔE_00_: Color change; H.F: subjected to hydrofluoric acid etching and silane coupling agent; S.B: subjected to sandblasting using AL_2_ O_3_; L.A: subjected to laser. ∙ Ctrl: not subjected to any surface treatment. ∙ S.B: subjected to sandblasting using AL_2_ O_3_. ∙ H.F: subjected to hydrofluoric acid etching and silane coupling agent. ∙ L.A: subjected to laser treatment

Color change (ΔE00) data were summarized in Table 1 and presented in Fig. 3. The effects of ceramic material, surface treatment, and their interaction on color change were statistically significant (*p* < 0.001), as shown in Tables 1 and 2. The significant interaction indicates that the effect of each surface treatment was not uniform across the two ceramic materials.

**Table 1:** Color change of different ceramic materials after different surface treatments

	Zirconia		Lithium Disilicate	
	Mean ± STD	95% CI	Mean ± STD	95% CI
Sandblasting (S.B)	3.36 ± 0.225^a^	(3.08–3.64)	5.97 ± 0.15^d^	(5.78–6.16)
Hydrofluoric Acid (H.F)	0.41 ± 0.103^b^	(0.28–0.53)	4.52 ± 0.34^e^	(4.09–4.94)
Laser (L.A)	2.48 ± 0.31^c^	(2.10–2.86)	1.04 ± 0.15^f^	(0.85–1.22)
*p*.value	*<0.001*		*<0.001*	

^*^Different superscript letters denote statistical significant difference

^*^Identical superscript letters denote statistical non-significant difference

^*^Data are presented as mean ± standard deviation

**Table 2:** Two-way ANOVA results for color change (ΔE_00_)

	Factors	Sum of Squares	df	Mean Square	F	*Sig*
Color Change (ΔE_00_)	Material	23.211	1	23.211	350.664	*˂0.001*
	Surface Treatment	46.077	2	23.039	348.053	*˂0.001*
	Material * Surface Treatment	41.380	2	20.690	312.576	*˂0.001*

df – degrees of freedom; ^*^statistically significant (*p* < 0.05)

The control group served as a reference group and was not included in inferential comparison of ΔE00 values, as no surface treatment was applied and no treatment-induced color change was anticipated.

For lithium disilicate, sandblasting produced the highest color change (5.97 ± 0.15), followed by hydrofluoric acid etching with silane (4.52 ± 0.34), while laser treatment resulted in the lowest and clinically acceptable color change (1.04 ± 0.15). In contrast, for zirconia, sandblasting caused the greatest color change (3.36 ± 0.23), followed by laser treatment (2.48 ± 0.31), whereas hydrofluoric acid etching with silane resulted in the lowest and clinically acceptable ΔE₀₀ (0.41 ± 0.10). These findings indicate that sandblasting consistently led to color changes beyond the clinically acceptable threshold for both materials (ΔE_00**=**_1.8 to 2.25) [35], while the effect of laser and hydrofluoric acid with silane was material-dependent.

**Fig. 4:**
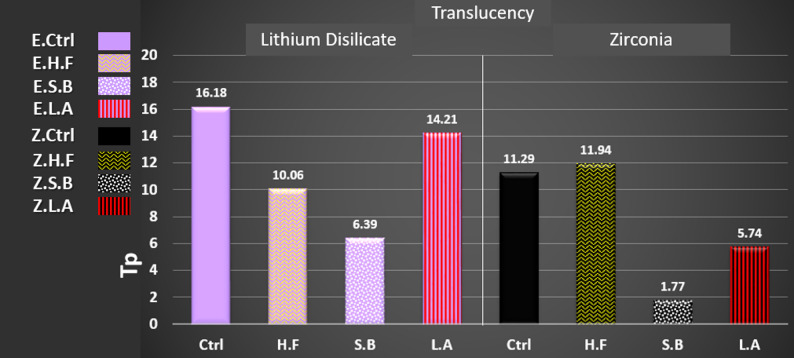
Bar graph presenting the translucency parameter of different subgroups. Tp: Translucency parameter; Ctrl: not subjected to any surface treatment; H.F: subjected to hydrofluoric acid etching and silane coupling agent; S.B: subjected to sandblasting using AL_2_O_3_; L.A: subjected to laser

Translucency parameter (Tp) data were summarized in Table 3 and presented in Fig. 4. The effect of both different ceramic materials and surface treatments on the translucency and their interaction was statistically significant (p = ˂0.001) as shown in Tables 3 and 4.

**Table 3:** Translucency pratmeter of different ceramic materials after different surface treatments

	Zirconia		Lithium Disilicate	
	Mean ± STD	95% CI	Mean ± STD	95% CI
Control (Ctrl)	11.29 ± 0.86^a^	10.22–12.35	16.18 ± 1.67^d^	14.11–18.25
Sandblasting (S.B)	1.77 ± 0.61^b^	1.01–2.52	6.39 ± 0.49^c^	5.79–7.00
Hydrofluoric Acid (H.F)	11.94 ± 1.16^a^	10.51–13.38	10.06 ± 0.63^a^	9.28–10.85
Laser (L.A)	5.74 ± 0.78^c^	4.77–6.72	14.21 ± 0.87^d^	13.14–15.29
*p*.value	*<0.001*		*<0.001*	

^*^Different superscribt letters denote statistical significant difference

^*^Identical superscribt letters denote statistical non-significant difference

^*^Data are presented as mean ± standard deviation

**Table 4:** Two-way ANOVA results for translucency (Tp)

	Factors	Sum of Squares	df	Mean Square	F	*Sig*
T.P	Material	162.212	1	162.212	143.61	*˂0.001*
	Surface Treatment	496.197	3	165.399	146.43	*˂0.001*
	Material * Surface Treatment	139.413	3	46.47	41.14	*˂0.001*

df – degrees of freedom; ^*^statistically significant (*p* < 0.05)

For lithium disilicate, the highest translucency was observed in the control samples (16.18 ± 1.67), followed by laser treatment (14.21 ± 0.87) and hydrofluoric acid etching with silane (10.06 ± 0.63), while sandblasting resulted in the lowest translucency (6.39 ± 0.49). For zirconia, hydrofluoric acid etching with silane produced the highest translucency (11.94 ± 1.16), followed by control samples (11.29 ± 0.86; not significantly different), laser treatment (5.74 ± 0.78), and sandblasting with the lowest value (1.77 ± 0.61). Statistically significant reductions in translucency were observed for sandblasting and laser treatment compared with controls in both materials (*p* < 0.001), indicating that surface treatment can markedly alter the optical properties of ceramics.

## Discussion

The null hypothesis was completely rejected, as both type of material and surface treatment exhibited significant effects on both color change and translucency of ceramic materials.

Recently, the revolution in dental ceramics in respect to microstructure, optical properties and mechanical properties offered wide range of indications; Moreover, the increase in demand and interest in achieving ultimate esthetic paved the way to the use of ceramic restorations in the esthetic zone [36]. 

Lithium disilicate was also chosen for this investigation because of its optical and strength characteristics. It is composed of needle-like glass ceramic crystals that comprises about two thirds of the glass ceramic volume. Because of the relatively low refractive index of lithium disilicate crystals, this material can be very translucent even with high crystalline content [37]. 

Zirconia restorations in its monolithic translucent forms have found appeal in prosthetic dentistry [38]. Polychromatic multilayered zirconia has been considered a great add to the library of esthetic monolithic restorations [39]. Additionally, the translucency of monolithic zirconia has significantly improved due to the increase in cubic concentration [40, 41]. Thus, combining the increasing in cubic content together with multilayering would be expected to reveal a restoration with highly appealing esthetics.

The choice of the thickness of the produced discs in this study relied on the minimum thickness recommended by the manufacturer for crowns in the esthetic zone including the premolar region. 1 mm thickness was specified for group UTML. Also, 10 mm was the standardized disc diameter to allow for the inclusion of all the layers of UTML in the sample [42]. 

Different surface treatment methods have been applied to monolithic restorations. Clinical success is significantly influenced by the characteristics of surface treatments applied to ceramic surfaces before cementation. The optical characteristics of ceramics may also be impacted by surface texture changes, and surface waviness has been shown to have strong correlation coefficients with optical parameters [43]. However, there is no conclusive evidence in literature supporting one method over the other when it comes to the effect of these treatments on the color and translucency properties of monolithic restorations [44, 45]. 

Hydrofluoric (HF) acid is an inorganic acid capable of etching glass and removing oxides from metals. It is typically used at a 4% to 10% concentration, which is considered safe for dental procedures, including the intraoral repair of restorations. During etching, the glassy matrix is selectively eliminated, exposing the crystalline phase and resulting in surface roughness. Consequently, etching procedures are employed to increase surface area and wettability to facilitate mechanical interlocking. However, hydrofluoric acid etching of zirconia remains controversial because of the predominantly crystalline phase of the material. Different concentrations, etching times, and temperatures of hydrofluoric acid can cause micromorphological changes and increase the roughness of the zirconia surface [24]. 

The Er: YAG laser parameters used included an output energy intensity of 200 mJ. This intensity is more reliable for zirconia surface treatment compared to 400 and 600 mJ, as higher intensities cause deep cracks in the zirconia surface and an extreme loss of mass. The Er: YAG application period used in the current study was 10 s, as this period has been reported to cause surface roughness without inducing surface cracks [28]. 

The IsoMet 4000 low speed precision saw was used in this study to cut materials with minimal sample deformation and low kerf loss. For standardization, all the specimens were manipulated by the same operator according to the manufacturers’ recommendations [19]. 

The clinical acceptability threshold (AT) for (ΔE_00_) defines the point at which observers accept a color difference between two items, typically in dentistry. It is generally accepted that a ΔE_00**≤**_1.8 is excellent, while values between 1.8 and 2.8 are often considered clinically acceptable in dental studies. Values above 5 are usually deemed unacceptable in dermatological matching [46]. 

The control group served as a reference group and was not included in inferential comparison of ΔE00 values, as no surface treatment was applied and no treatment-induced color change was anticipated.

When the present results are interpreted against previous literature, the magnitude of the optical change becomes more clinically meaningful. In the current study, lithium disilicate demonstrated a ΔE00 of 5.97 after sandblasting, whereas zirconia showed a substantially lower response. This value is markedly higher than the mild changes reported after less aggressive airborne-particle abrasion protocols. Salem and Mohsen reported ΔE values of only 0.89 for sandblasted IPS e.max CAD compared with 0.74 for untreated controls after 50 μm Al₂O₃ abrasion, indicating that modest surface treatment may produce statistically detectable but clinically limited color alteration [47]. Likewise, Soares et al. found that surface treatment significantly influenced the color stability and translucency of lithium disilicate, but the reported changes remained far below the severe level observed in the present investigation [20]. Therefore, the present ΔE00 value could be interpreted as an outlying but plausible response under a more aggressive protocol, likely related to the use of larger abrasive particles and the greater surface damage they may generate. Rather than contradicting previous studies, the current findings may represent the upper end of the response spectrum when abrasion intensity increases.

The different behavior between lithium disilicate and zirconia could be explained by their fundamentally different crystal morphology and light-transport mechanisms. Lithium disilicate is a glass-ceramic composed of elongated interlocking needle-like crystals embedded in a glassy matrix. Its esthetic performance depends heavily on controlled light transmission through this silica-based matrix; therefore, disruption of the surface or subsurface structure might have markedly increased scattering and sharply altered color coordinates. In contrast, 5Y-PSZ zirconia is a polycrystalline ceramic with no glass phase and contains a substantial cubic fraction that provides intrinsic translucency through reduced birefringence and more uniform grain optics. Although sandblasting still roughens zirconia and reduces translucency, its optical response might have been less dramatic because there was no glass matrix to be fractured or preferentially abraded. This interpretation is consistent with comparative optical studies showing that 5Y zirconia exhibited translucency values intermediate between conventional zirconia and lithium disilicate, while maintaining a different scattering behavior derived from its grain-based microstructure [48]. Accordingly, the greater ΔE00 observed for lithium disilicate in the present study could be attributed to the higher sensitivity of glass-ceramic microstructures to abrasive surface trauma when compared with highly translucent zirconia.

The result of this study showed that sandblasting induced a greater color change than hydrofluoric acid etching with silane application in the lithium disilicate samples but both surface treatments led to color change beyond the clinical acceptable range, while laser treatment showed color change within the clinical acceptable range. Also, the results showed that control samples and laser treatment had the highest translucency with no significant difference between them while there was significant difference in translucency between the control samples versus the sandblasted and the hydrofluoric acid etching with silane application samples.

This finding could be attributed to the fact that sandblasting with 50 μm Al₂O₃ particles might have produced irregular and uneven surface topographies at varying depths [49]. In contrast, IPS e.max CAD is composed of two crystalline phases: a primary phase of elongated lithium disilicate crystals and a secondary phase of lithium orthophosphate, both embedded within a glass matrix [50, 51]. With hydrofluoric acid etching, this may be attributed to that it selectively dissolved the glass matrix and the lithium orthophosphate phase, thereby exposing the lithium disilicate crystals and generating a rough, irregular surface morphology. Conversely, laser surface treatment might have induced thermal vaporization of the substrate through absorption of laser energy, which was likely converted into thermal energy, producing thermo-mechanical effects on the ceramic surface [51]. Laser irradiation appeared to have resulted in fusion and melting of the most superficial ceramic layer, thereby modifying the surface characteristics [51, 52].

Possible alterations in surface texture and roughness resulting from the applied surface treatments could significantly influence the optical properties of ceramic materials. A smooth surface promotes specular light reflection; whereby incident light is reflected uniformly in a single direction [53]. In contrast, a rough surface results in diffuse reflection, with light scattered in multiple directions. The perceived color of any object is therefore influenced by its intrinsic optical properties, including its ability to transmit, reflect, and absorb light [54]. When most of the incident light is scattered and reflected, the material appears opaque. Conversely, when only a portion of the light is scattered and the majority is transmitted, the material appears translucent [55]. Sandblasting and hydrofluoric acid etching might have reduced the translucency of the ceramic materials by removing portions of the glassy matrix, thereby decreasing light transmission through the ceramic [56, 57]. This could explain why the sandblasting and hydrofluoric acid demonstrated higher values of ∆E.

The result of this study showed that sandblasting had more color change than the laser treatment in the zirconia samples but both surface treatments led to color change beyond the clinical acceptable range, while hydrofluoric acid etching with silane application showed color change within the clinical acceptable range. Also, the results showed that control samples and the hydrofluoric acid etching with silane application treatment had the highest translucency with no significant difference between them while there was significant difference in translucency between the control samples versus the sandblasted and the laser treated samples.

It has been reported that airborne particle abrasion with 50–110 μm alumina particles could effectively roughen and modify the surface topography of zirconia, leading to increased light scattering and subsequent alterations in its optical properties [58, 59]. 

This in agreement with Kim and Ahn [60] who explained that the sandblasting impact process transferred substantial kinetic energy and heat to the alumina particles, which might have caused local surface melting and microcracks on the zirconia surface, subsequently resulting in alterations to its optical properties [61]. 

Laser treatment might have removed surface particles through micro-explosions and vaporization—a process known as ablation—which could have resulted in changes in color [62]. This results in agreement with Vohra et al. [56].

Hydrofluoric acid etching produced insufficient surface modifications because it might selectively dissolve the glassy matrix of glass-based ceramics but is unable to adequately roughen the dense polycrystalline structure of zirconia. Moreover, zirconia is characterized by high density, small crystal size, and high hardness, which further limit the effectiveness of this etching approach [63, 64]. The optical changes that occurred could be related to this limited surface interaction rather than true chemical etching of the zirconia. A possible contributing factor might have been the larger grain size of cubic-phase zirconia, which has been reported to enhance diffusion pathways and diffusion-related chemical alterations. Kolakarnprasert et al. reported larger grain dimensions in cubic zirconia, while Kosmač et al. suggested that increased grain size could facilitate acid diffusion and diffusion-related alterations, with possible superficial interaction with pigment oxides or surface-associated components [65, 66]. 

This is in agreement with Zarone et al. [67], who studied the effect of hydrofluoric acid etching on feldspathic, alumina and zirconia ceramic and showed that hydrofluoric acid etching had no significant effect on the surface of zirconia ceramic.

## Conclusions

Both ceramic type and surface treatment significantly influenced the optical properties of lithium disilicate and zirconia. Sandblasting caused the greatest color change and the largest reduction in translucency in both materials, consistently exceeding clinically acceptable limits. Laser treatment preserved acceptable color stability in lithium disilicate, while hydrofluoric acid with silane produced the lowest and clinically acceptable color change in zirconia. Translucency was highest in untreated lithium disilicate, and in zirconia both hydrofluoric acid–treated and untreated samples showed the greatest translucency.

### Clinical recommendations

The choice of surface treatment should be material-specific, as it can markedly alter the esthetic performance of ceramic restorations.

Since sandblasting produced the greatest color change and the largest reduction in translucency, its use on visible surfaces should be minimized, especially in anterior restorations.

For lithium disilicate, laser treatment showed the most favorable optical performance and may be considered a conservative option when clinically indicated. Hydrofluoric acid with silane may still be used for bonding purposes, although greater color change was observed.

For zirconia, less aggressive surface treatments better preserved optical properties than sandblasting. Therefore, airborne-particle abrasion should be restricted to cases requiring mechanical retention and performed using the mildest effective parameters.

### Limitations of this study

Certain limitations of this study should be acknowledged. First, as an in vitro investigation with a limited sample size, the findings may not fully reflect clinical scenarios. Second, the study did not incorporate aging simulations or surface analyses, such as X-ray diffraction and surface roughness measurements. Finally, the prolonged 9.5% HF immersion protocol was strictly experimental and should not be interpreted as a clinically recommended zirconia surface treatment.

## Data Availability

The datasets generated and analysed during the current study are available from the corresponding author on reasonable request.

## Abbreviations

CAD-CAM: Computer aided design and computer aided manufacturing
STL: Standard tessellation language
Tp: Translucency parameter
